# Validity of parental recalls to estimate vaccination coverage: evidence from Tanzania

**DOI:** 10.1186/s12913-018-3270-z

**Published:** 2018-06-13

**Authors:** Peter Binyaruka, Josephine Borghi

**Affiliations:** 1 Ifakara Health Institute, Box 78373, Dar es Salaam, PO Tanzania; 2 London School of Hygiene and Tropical Medicine, 15-17 Tavistock Place, WC1H 9SH, London, UK

**Keywords:** Vaccination, Immunisation, Validity, Parental recall, Recall bias, Vaccination card, Tanzania

## Abstract

**Background:**

The estimates of vaccination coverage are measured from administrative data and from population based survey. While both card-based and recall data are collected through population survey, and the recall is when the card is missing, the preferred estimates remain of the card-based due to limited validity of parental recalls. As there is a concern of missing cards in poor settings, the evidence on validity of parental recalls is limited and varied across vaccine types, and therefore timely and needed. We validated the recalls against card-based data based on population survey in Tanzania.

**Methods:**

We used a cross-sectional survey of about 3000 households with women who delivered in the last 12 months prior to the interview in 2012 from three regions in Tanzania. Data on the vaccination status on four vaccine types were collected using two data sources, card and recall-based. We compared the level of agreement and identified the recall bias between the two data sources. We further computed the sensitivity and specificity of parental recalls, and used a multivariate logit model to identify the determinants of parental recall bias.

**Results:**

Most parents (85.4%) were able to present the vaccination cards during the survey, and these were used for analysis. Although the coverage levels were generally similar across data sources, the recall-based data slightly overestimated the coverage estimates. The level of agreement between the two data sources was high above 94%, with minimal recall bias of less than 6%. The recall bias due to over-reporting were slightly higher than that due to under-reporting. The sensitivity of parental recalls was generally high for all vaccine types, while the specificity was generally low across vaccine types except for measles. The minimal recall bias for DPT and measles were associated with the mother’s age, education level, health insurance status, region location and child age.

**Conclusion:**

Parental recalls when compared to card-based data are hugely accurate with minimal recall bias in Tanzania. Our findings support the use of parental recall collected through surveys to identify the child vaccination status in the absence of vaccination cards. The use of recall data alongside card-based estimates also ensures more representative coverage estimates.

## Background

In an effort to achieve the sustainable development goal three (SDG 3) and further reduce under-five mortality and morbidity [1], effective provision of lifesaving childhood immunisations is urgently needed [2–4]. Immunisation is one of the most important public health interventions, in terms of potential health impact and cost effectiveness, and has been universally recommended [5]. It is therefore important to track and monitor progress, and evaluate immunisation programs such as the Expanded Programme on Immunisation (EPI) and the Global Alliance for Vaccines and Immunisations (GAVI) by estimating vaccination coverage through routine and/or any other reliable measurements. Vaccination coverage estimates are important for planning, priority setting, and implementing interventions to improve population health [4, 6–8].

There are two main methods of estimating vaccination coverage [4, 9]: provider administrative records as routinely collected information during service delivery, and population-based household surveys such as Multiple Indicators Cluster Surveys (MICS) [10] and Demographic and Health Surveys (DHS) [11]. Each method has pros and cons [8]. In low- and middle- income countries (LMICs) for example, coverage based on administrative data has the advantage of being routinely available [12, 13], but its reliability has been questioned due to bias (inaccuracy in population denominators, registration, or reporting) [2, 4, 9, 14] and missing data on community outreach and national vaccination day campaigns. Vaccination coverage based on population surveys are based on valid population denominators [8, 15], but they cover only a sampled population and surveys are not carried out routinely due to resource constraints [12]. Population surveys have also been used as an independent check on coverage levels estimated through routine administrative data [12, 16].

Vaccination coverage is commonly assessed within household surveys with reference to vaccination cards and/or parental recall [4, 16]. Although data from vaccination cards are considered more reliable than recall, both approaches are prone to selection bias (use of an inaccurate sampling frame) and information bias (inaccurate data from respondents) [8, 17].

The absence of vaccination cards within households is a frequent issue in LMIC settings. For example, more than half of mothers surveyed in India [18] and Sudan [19] did not have vaccination cards; about one-third of mothers in Bangladesh had no cards [20]; and almost one-third of households on average did not have cards according to population surveys across 101 countries, mostly in LMICs [21]. Given this, in LMICs the estimated vaccination coverage through population surveys may be incomplete where cards are missing, and data from parental recall provides an alternative in such cases.

Evidence on the validity of parental recall for measuring vaccination coverage in relation to vaccination cards or routine administrative data is limited in LMICs, especially in Sub-Saharan Africa. For example, when compared to administrative data, parental recall was generally found to underestimate vaccination coverage across vaccine types [4, 7, 22, 23]. On the other hand, when compared to data from vaccination cards, parental recall was found to be relatively consistent overall with slight variation across vaccine types and settings [19, 20, 24–26]. However, these studies are dated, with no studies being carried out in the last 10 years. In addition, among the available studies only two were in Sub-Saharan Africa, and their focus has been on measles and diphtheria-tetanus-pertussis (DPT) vaccine types, with less attention to other vaccines. To date there has been no study from East Africa, although missing vaccination cards are a concern in these countries. Since population surveys are commonly conducted to estimate vaccination coverage in LMICs, it is important to assess whether parental recall is a valid measure of coverage that could supplement card-based estimates where cards are missing to provide coverage estimates. Further, there has been very limited attention to the determinants or factors related with recall bias for vaccination coverage, with few exceptions such as [19, 20, 26].

In this study, we assessed the validity of vaccination coverage data based on parental recall, as compared to data from vaccination cards, and identified the individual and household-level determinants of recall bias in Tanzania. We used data from a cross-sectional household survey (for households with a child of less than 12 months of age) capturing data from vaccination cards and from parental recall based on four vaccine types: Bacillus Calmette–Guérin (BCG) vaccine, oral polio vaccine (OPV), diphtheria-tetanus-pertussis (DTP) vaccine, and measles vaccine. The BCG vaccine and the first doses of OPV and DPT are administered after birth, while the first dose of measles vaccine is administered after nine months. Our household survey data were selected in preference to national DHS data as the latter only ask for parental recall when the vaccination card or its data are missing.

## Methods

### Study setting

This study was conducted in 11 districts from three regions (i.e. Pwani, Morogoro and Lindi) out of 30 regions in Tanzania. The population of Pwani region is just above a million, over two million in Morogoro region, and less than a million in Lindi region [27]. The data were derived from a baseline survey done as part of an impact evaluation of a payment for performance (P4P) programme in Pwani region [28, 29]. The survey was done in all districts of Pwani region and in four districts from Morogoro and Lindi region (Morogoro rural, Morogoro urban, Mvomero, and Kilwa). The four districts from Morogoro and Lindi region were selected for comparison purpose such that they were geographically contiguous to districts in Pwani and similar in relation to indicators of: poverty, literacy rates, rate of institutional deliveries, infant mortality, population per health facility, and the number of children under 1 year of age per capita [29]. Comparison districts were also free from programmes to improve maternal and child health, which could confound the evaluation of P4P.

### Data sources and sampling

Data were collected from a cross-sectional household survey across all 11 districts in the three regions that were survey. Health facilities were the primary sampling unit. We included all 6 hospitals and 16 health centres that were eligible for inclusion in P4P, and randomly sampled 53 dispensaries from Pwani region. An equivalent number of facilities in comparison districts were sampled by facility level of care. In total, we included equal number of facilities between study arms; i.e. 75 facilities from Pwani region (intervention arm) and 75 facilities from Morogoro and Lindi (comparison arm). We also randomly sampled 20 households of women aged (15–49 years) who had delivered in 12 months prior to the survey from the catchment area of each health facility [29]. In total, we aimed to surveyed 3000 households with eligible women from across the three regions. The sampling strategy of facilities and households is detailed elsewhere [29].

### Data collection

The household survey was carried out in January and February 2012. Trained field researchers administered a structured questionnaire to the household head and to an eligible woman with a child of less than 12 months of age. The survey questionnaire was adapted from the World Bank Impact Evaluation Toolkit [30]. The household questionnaire captured information on household background characteristics (e.g. ownership of assets) to assess the household’s socioeconomic status. The women’s questionnaire captured data on background characteristics of women, and data on service utilisation for maternal and child health services, including childhood vaccination status.

### Measures of vaccination status

Four vaccination types were considered: BCG vaccine, OPV, DTP vaccine, and measles vaccine. Vaccination status was obtained by reviewing the vaccination card where available and through recording the appropriate vaccine type and number of doses received. All parents were also asked to recall the vaccination status of their child for any dose across vaccine types. However, the validity of parental recall was assessed among parents with both information on recall and vaccination card. Parental recall was assessed mainly in terms of vaccination coverage rather than number of doses.

### Data analysis

We first calculated vaccination coverage levels from vaccination cards and parental recalls across all vaccine types considered. To test whether parental recall over- or under-estimated vaccination coverage relative to cards, we compared the level of agreement in coverage levels derived from each source, for those observations that contained estimates from both sources: recall and vaccination card.

We compared parental recall to card-based data, assuming the latter to be accurate. Recall bias was defined as the discrepancy in vaccination status between the two data sources (i.e. false positives and false negatives). We then disaggregated the recall bias into whether parental recall over-reported (recalled as vaccinated while not) or under-reported (recalled as not–vaccinated while vaccinated). We further computed the level of agreement between the estimates from the two data sources, and estimated the sensitivity and specificity of parental recall based on the two-by-two table (Table 1):

**Table 1:** Two-by-two table for calculating sensitivity and specificity

Agreement of sources		Vaccination status (Card-based source)		
		YES	NO	
Vaccination status (Recall-based source)	YES	True positives (TP)	False positives (FP)	TP + FP
	NO	False negatives (FN)	True negatives (TN)	FN + TN
		TP + FN	FP + TN	Total (N)

The level of *agreement/concordance* is the percentage of children whose parents accurately recalled the vaccination status of their children ([TP + TN]/Total). *Sensitivity of recall* is the percentage of children whose parents recalled they were vaccinated and they were vaccinated according to their vaccination cards (TP/[TP + FN]). *Specificity of recall* is the percentage of children whose parents recalled they were unvaccinated and they were not vaccinated according to their vaccination cards (TN/[FP + TN]).

We also calculated Kappa statistics for each type of vaccine as an alternative assessment of the degree of agreement between two data sources. The Kappa statistic is a measure of reliability that takes into account the agreement expected on the basis of chance [31, 32]. A Kappa statistic ≤0.20 shows slight to poor agreement, 0.21–0.40 shows a fair agreement, 0.41–0.60 moderate agreement, 0.61–0.80 substantial agreement, and 0.81–1.00 indicates almost perfect agreement [33]. However, the Kappa statistic is commonly affected by the prevalence of an indicator and bias/ level of disagreement which leads to a trade-off paradox (high agreement but low Kappa) [33, 34]. In response, T Byrt, J Bishop and JB Carlin [35] proposed the use of Prevalence and Bias Adjusted Kappa (PABAK) to avoid the paradox. This study applied both approaches of estimating Kappa statistics.

To identify the determinants of parental recall bias, we applied a series of multivariate logit regression models by vaccine types. Our binary dependent variable for recall bias took the value of 1 if the two data sources disagreed and 0 otherwise for a given vaccine. A set of individual and household characteristics were included in the model as potential determinants of recall bias. These included child age (in months), maternal age (in years), marital status, health insurance status, religion, maternal education, occupation, number of births/parity, place of residence (rural/urban district), and household wealth status in quintiles. The wealth quintiles were generated from wealth scores derived by the principal component analysis based on 42 items of household characteristics and asset ownership [36, 37]. All analyses were performed using STATA version 13.

## Results

Although we sampled 3000 households, only 2882 were interviewed (i.e. 96% response rate). Out of 2882 households interviewed in 2012, the majority of them 2816 (97.7%) reported having received a child vaccination card from the health facility, and 2460 (85.4%) were able to present the vaccination card during the survey. The average age of children with vaccination cards was 8 months, and that of their mothers was 26 years (Table 2). Most mothers were married, uninsured, Muslim, with primary or above education, and located in rural districts.

**Table 2:** Individual and household characteristics of women with vaccination cards (*n* = 2460)

Characteristics	Description	Mean [SD]	Mean in %
*Individual-level*			
Child age	Mean child age (0–11) months	8.1 [2.9]	
Maternal age	Mean maternal age (15–49) years	26.3 [6.6]	
Marital status	Married women		67.5
Health insurance status	Insured women		8.2
Religion	Muslim women		76.3
Education	Women with no education		19.7
Occupation	Women doing farming activities		50.1
Parity	Mean number of births	2.6 [1.7]	
*Household-level*			
Place of residence	Household in rural district		82.4
Place of residence	Household in Pwani region		47.5
Wealth quintile 1	Poorest household		21.2
Wealth quintile 2	Poor household		19.4
Wealth quintile 3	Middle wealth household		19.7
Wealth quintile 4	Least poor household		19.6
Wealth quintile 5	Least poorest household		20.1

Notes: *SD* Standard Deviation; all variables presented with percentage mean are dummy variables

**Fig. 1:**
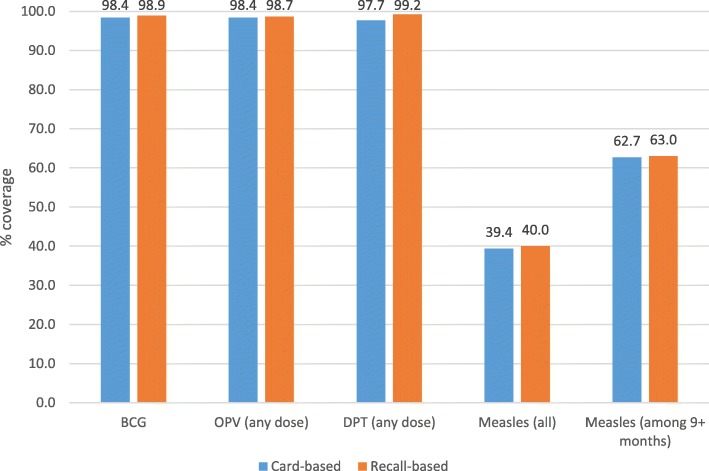
Vaccination coverage levels for under one year children from two data sources

Overall vaccination coverage levels from the two data sources were generally similar (Fig. 1). However, the recall data resulted in slightly higher coverage estimates relative to vaccination cards. Most children (> 97%) received BCG, at least one dose of OPV and DPT, and about two-thirds of children aged 9 months or more received measles vaccine. However, when all children under a year of age were considered, the coverage for measles vaccine was about 40% (95% CI: 38.1–41.9%) from recall and 39% (95% CI: 37.4–41.3%) from vaccination cards.

The level of agreement between parental recall compared to cards was above 94% across vaccine types (Table 3), with minimal parental recall bias. The highest level of recall bias was for measles, that is about 5.8% (Table 3). Recall bias due to over-reporting was slightly higher than that due to under-reporting. The sensitivity of parental recall was generally high for all vaccine types, while the specificity was generally low across vaccine types except for measles (Table 3). The unadjusted Kappa statistics revealed a trade-off paradox, because the Kappa values were low for vaccines with higher level of agreement and vice versa. In contrast, the adjusted Kappa values showed perfect agreement between the two data sources with values above 0.87 for all vaccine types [31]. Adjusted Kappa values were also consistent with the prior level of agreement and recall bias, that is relatively lower for measles vaccine than other vaccine types (Table 3).

**Table 3:** Measures of agreement between two data sources (card- vs recall-based)

	BCG	OPV	DPT	Measles	Measles (9+ months)
	(*n* = 2460)	(*n* = 2460)	(*n* = 2460)	(*n* = 2460)	(*n* = 644)
Agreement/concordance (%)	98.0	97.7	97.7	94.2	94.4
Parental recall bias (%)	2.0	2.3	2.3	5.8	5.6
Over-reporting (%)	1.3	1.3	1.9	3.2	3.0
Under-reporting (%)	0.7	1.0	0.4	2.6	2.6
Sensitivity (%)	99.3	98.9	99.6	93.4	95.8
Specificity (%)	22.5	20.0	16.1	94.6	92.1
Kappa statistic (unadjusted)	0.26	0.21	0.23	0.88	0.88
Kappa statistic –PABAK	0.96	0.95	0.95	0.88	0.89

Notes: Prevalence and Bias Adjusted Kappa (PABAK) = (2*P*_*O*_ -1), where *P*_*O*_ indicates proportion of observed agreement

Parental recall bias for BCG and OPV was invariant across individual and household characteristics (Table 4). In contrast, recall bias for DPT was greater for parents with younger children, for older mothers, and for those with insurance. Those with low levels of education and from Pwani region had higher chance of recall bias for measles (Table 4). Specifically, recall bias for DPT reduced by 12% for each additional month of child age, and increased by 6% with each additional year of maternal age. Mothers who were insured were almost two times more likely to inaccurately recall DPT vaccination status than uninsured mothers. Furthermore, mothers with no education were almost three times more likely than educated mothers to inaccurately recall measles vaccination status. Recall errors for measles vaccine were twice as likely among mothers residing in Pwani region than those in other regions. The results in Table 4 remained unchanged when the regression analyses were adjusted for clustering at the facility level (data not shown).

**Table 4:** Multivariate Logit regression model results on the determinants of parental recall bias by vaccine types

Characteristics/determinants	Vaccine types			
	BCG	OPV	DPT	Measles
	OR (*p*-value)	OR (*p*-value)	OR (*p*-value)	OR (*p*-value)
*Individual-level*				
Child age (months)	0.93 (0.172)	0.95 (0.307)	0.88 (0.006)***	1.28 (0.247)
Maternal age (years)	1.02 (0.491)	1.02 (0.423)	1.06 (0.049)**	1.05 (0.233)
Married	1.25 (0.518)	0.80 (0.466)	1.51 (0.240)	0.61 (0.205)
Insured	0.67 (0.520)	1.14 (0.763)	2.09 (0.047)**	1.84 (0.256)
Muslim	1.84 (0.160)	1.87 (0.120)	1.48 (0.313)	1.67 (0.300)
No education	0.51 (0.137)	0.86 (0.664)	0.96 (0.904)	2.76 (0.019)**
Farmer	1.30 (0.468)	1.33 (0.392)	1.16 (0.647)	0.75 (0.486)
Parity (# of births)	1.02 (0.874)	1.19 (0.104)	0.94 (0.563)	0.81 (0.219)
*Household-level*				
Rural households	0.66 (0.300)	0.69 (0.304)	0.91 (0.807)	1.25 (0.655)
Pwani households	0.76 (0.394)	1.12 (0.692)	1.50 (0.176)	2.48 (0.018)**
Quintile 1-poorest	1.49 (0.447)	0.75 (0.559)	0.74 (0.556)	0.56 (0.362)
Quintile 2-poor	0.51 (0.285)	0.85 (0.738)	1.09 (0.853)	0.36 (0.115)
Quintile 3-middle	1.01 (0.993)	0.51 (0.183)	0.37 (0.077)*	0.96 (0.940)
Quintile 4-least poor	0.60 (0.361)	0.56 (0.239)	0.89 (0.808)	0.69 (0.516)
Quintile 5-least poorest (reference)				
Observations (N)	2324	2324	2324	634

Notes: *OR* Odds Ratio; The estimation for measles vaccines was restricted to children with at least 9 months of age; OPV and DPT vaccination status considered at least one dose; The dependent variable (recall bias) took the value of 1 if the two data sources disagree and 0 otherwise; These results remain unchanged when adjusted for clustering at the facility level in the analysis; *** denotes significance at 1%, ** at 5%, and * at 10% level

## Discussion

This study assessed the validity of parental recall against vaccination cards for estimating vaccination coverage using a population survey in Tanzania. We used card-based data as the reference as these data are commonly preferred to recall data in population surveys such as the DHS [11]. To this end, we compared the two data sources and quantified the level of agreement, recall bias, and identified the determinants of recall bias. Both sources of data estimated higher coverage levels for BCG, OPV and DPT, compared to measles vaccine, reflecting the age of our sample (average 8 months), with measles vaccine typically being administered from 9 months. Our study found that the discrepancy in coverage levels between the two data sources was minimal with limited recall bias (< 6%). Recall bias was greatest for measles vaccine. The sensitivity of parental recall was generally high whereas its specificity was low except for measles. Factors such as maternal age, education, location and age of the child were significantly associated with recall bias for DPT; while the level of education and region of residence significantly associated with recall bias for measles.

Our finding that vaccination coverage was slightly overestimated with a recall-based compared to a card-based approach is similar to findings from other studies [19, 24]. The overestimation through recall may possibly be explained by social desirability bias, especially when child vaccination practice is considered socially desirable [38]. The high level of agreement between recall and card-based data is similar to that reported in Egypt [24], Sudan [19], Bangladesh [20] and India [25]. In the above studies, the accuracy of recall was more than 80% for measles and ranged from 61 to 98% for BCG and DPT. In Costa Rica, the recall and card-based data correlated at 71% [26].

The high sensitivity and specificity of recall for measles vaccine could be due to the level of detail provided in the respective question in the survey. The survey question for measles vaccine was different from others, as respondents were told specifically that the measles vaccine is given at the age of nine months and above. Indeed, data collection techniques are known to affect levels of recall bias [38]. There should be a need to explicitly specify age limits in all survey questions that seeks parental recall for vaccine types. Previous studies in LMICs have reported both high sensitivity and specificity of parental recall across vaccines of at least 80 and 67% respectively [19, 20]. The high sensitivity and specificity suggests that the recall can largely identify correctly the vaccination status of being vaccinated and of being unvaccinated respectively [39].

We also found that parents with younger children were more likely to demonstrate recall bias for DPT than those with older children, similar to that reported in Cost Rica [26]. Our finding that younger mothers were less likely to be subject to recall bias is consistent with findings from Bangladesh [20]. The findings based on age partly suggests the following: first, the survey which seeks parental recall on child vaccination should follow women with slightly older children to improve the accuracy in recall; and the sample of older women with experience in child bearing should be reduced to minimise the risk of recall bias. The finding of greater accuracy on recalling measles vaccine status among educated mothers is also consistent with findings from Bangladesh [20], but contrary to findings in Sudan [19]. A study by SS Coughlin [38] revealed that factors like age, education and socioeconomic status of the respondents, affected the accuracy of recall.

Our findings have important implications for vaccination programmes in LMICs. In these settings, the quality of administrative data is typically poor, and the estimates from population household surveys are therefore being used for coverage estimation. As long as parental recall of vaccinations is accurate, it can be used to supplement card-based data in situations where cards are missing. Our findings support the use of recall data alongside card-based estimates, in populations where missing cards are widespread, to ensure more representative coverage estimates. Although parental recall could serve as an additional source of data, efforts are also needed to encourage high card-retention rate with complete and reliable information documented since we found almost 12.3% received the cards but were unavailable during the survey. To encourage high card-retention rate can be through educating mothers on the importance of keeping the vaccination cards or any other medical records. In the case of not being given any vaccination card, health providers could be encouraged to routinely record, document and handle medical cards to all attended clients. Moreover, more efforts are needed to strengthen the administrative data which are routinely collected and relatively less expensive and providing a much more complete time series compared to population based surveys which are only carried out every few years. This study also indicates that recall bias was generally random across the surveyed population, with few systematic associations between bias and parental education, age, health insurance status and child age.

This study has the following limitations. First, we assessed the validity of parental recall by assuming card-based data were the reference, but these data are sometimes incomplete or inaccurate. While studies in high-income countries have used administrative data as a benchmark to validate parental recalls [4], we were unable to use these data in our setting due to its limited completeness and reliability at the time of the study (i.e. inaccuracy in population denominators, registration, or reporting) [2, 4, 9, 14] compared to card-based data. Second, our assessment was based on a sample of children under one-year age as opposed to most vaccination studies which uses children aged 12–23 months. However, our main goal was to compare the accuracy on vaccination status between two data sources rather than assessing the coverage estimates, since the later might be underestimated when using younger children. Nevertheless, further validation among older children is important given that recall bias is potentially affected by a recall time interval [38]. Third, we used household data from a random sample of 20 households per facility to assess the validity of parental recall. It is quite possible that the sample of households did not accurately represent the entire population of women with children eligible for vaccination in the catchment area. Fourth, we were unable to compare the appropriate number of doses for DPT and OPV between data sources because the recall data were unable to distinguish the number of doses. The recall and coverage of BCG and measles vaccines were relatively easier to obtain compared to OPV and DPT which typically include three doses. Lastly, we were unable to validate the recall of parents without cards (i.e. 12.6%) as we restricted our comparison to parents with data from both sources, card and recall. However, it is conceivable that those with cards have more accurate recall than those without. A further concern is if parents with cards are systematically different to those without cards and that these differences affect recall. To validate the recalls for parents without cards, it needs to be compared to administrative data for example, and this is an important area for future research in our setting. However, if the cards are missing at random, which was the case in our dataset, then it seems reasonable to extrapolate the findings to households without cards.

## Conclusion

This study assessed the validity of parental recalls against card-based data in Tanzania, and found most of the children’s vaccination status across vaccine types were accurately identified through parental recall. The limited recall bias for DPT and measles were associated with the mother’s age, education level, health insurance status, region location and child age. Our findings support the use of parental recall collected through surveys to identify the child vaccination status in the absence of vaccination cards. However, further research is needed to validate these findings against routine administrative data.

## Abbreviations

BCG: Bacillus Calmette–Guérin
DHS: Demographic and Health Surveys
DPT: Diphtheria-tetanus-Pertussis
EPI: Expanded Programme on Immunisation
FN: False negatives
FP: False positives
GAVI: Global Alliance for Vaccines and Immunisations
LMICs: Low- and middle- income countries
MICS: Multiple Indicators Cluster Surveys
OPV: Oral Polio Vaccine
PABAK: Prevalence and Bias Adjusted Kappa
SD: Standard Deviation
SDG3: Sustainable Development Goal three
TN: True negatives
TP: True positives

## References

[1] United Nations (2016). The Sustain Dev Goals Report 2016.

[2] Lim SS, Stein DB, Charrow A, Murray CJ (2008). Tracking progress towards universal childhood immunisation and the impact of global initiatives: a systematic analysis of three-dose diphtheria, tetanus, and pertussis immunisation coverage. Lancet.

[3] WHO (2010). Monitoring the building blocks of health systems: A handbook of indicators and their measurement startegies.

[4] Miles M, Ryman TK, Dietz V, Zell E, Luman ET (2013). Validity of vaccination cards and parental recall to estimate vaccination coverage: a systematic review of the literature. Vaccine.

[5] Bos E, Batson A. Using immunization coverage rates for monitoring health sector performance. Washington, DC: The World Bank; 2000.

[6] Burton A, Monasch R, Lautenbach B, Gacic-Dobo M, Neill M, Karimov R, Wolfson L, Jones G, Birmingham M (2009). WHO and UNICEF estimates of national infant immunization coverage: methods and processes. Bull World Health Organ.

[7] Luman ET, Ryman TK, Sablan M (2009). Estimating vaccination coverage: validity of household-retained vaccination cards and parental recall. Vaccine.

[8] Cutts FT, Claquin P, Danovaro-Holliday MC, Rhoda DA (2016). Monitoring vaccination coverage: defining the role of surveys. Vaccine.

[9] Murray CJ, Shengelia B, Gupta N, Moussavi S, Tandon A, Thieren M (2003). Validity of reported vaccination coverage in 45 countries. Lancet.

[10] Statistics and Monitoring: Multiple Indicator Cluster Survey. Available: http://www.unicef.org/statistics/index_24302.html [http://www.unicef.org/statistics/index_24302.html]. Accessed 31 May 2018.

[11] MEASURE DHS Demographic and Health Surveys. Available: http://www.measuredhs.com/ [http://dhsprogram.com/]. Accessed 31 May 2018.

[12] Borgdorff MW, Walker GJ (1988). Estimating vaccination coverage: routine information or sample survey?. J Trop Med Hyg.

[13] Cutts FT, Waldman RJ, Zoffman HM (1993). Surveillance for the expanded Programme on immunization. Bull World Health Organ.

[14] Haddad S, Bicaba A, Feletto M, Fournier P, Zunzunegui MV (2010). Heterogeneity in the validity of administrative-based estimates of immunization coverage across health districts in Burkina Faso: implications for measurement, monitoring and planning. Health Policy Plan.

[15] Cutts FT, Izurieta HS, Rhoda DA (2013). Measuring coverage in MNCH: design, implementation, and interpretation challenges associated with tracking vaccination coverage using household surveys. PLoS Med.

[16] WHO (2005). Immunization coverage cluster survey—reference manual.

[17] Eisele TP, Rhoda DA, Cutts FT, Keating J, Ren R, Barros AJ, Arnold F (2013). Measuring coverage in MNCH: total survey error and the interpretation of intervention coverage estimates from household surveys. PLoS Med.

[18] Ramakrishnan R, Venkata Rao T, Sundaramoorthy L, Joshua V (1999). Magnitude of recall bias in the estimation of immunization coverage and its determinants. Indian Pediatr.

[19] Gareaballah ET, Loevinsohn BP (1989). The accuracy of mother's reports about their children's vaccination status. Bull World Health Organ.

[20] Selimuzzaman A, Ullah M, Haque M (2009). Accuracy of mothers’ reports regarding vaccination status of their children in urban Bangladesh. TAJ: Journal of Teachers Association.

[21] Brown J, Monasch R, Bicego G, Burton A, Boerma JT (2002). An assessment of the quality of national child immunization coverage estimates in population-based surveys.

[22] Bolton P, Holt E, Ross A, Hughart N, Guyer B (1998). Estimating vaccination coverage using parental recall, vaccination cards, and medical records. Public Health Rep.

[23] Suarez L, Simpson DM, Smith DR (1997). Errors and correlates in parental recall of child immunizations: effects on vaccination coverage estimates. Pediatrics.

[24] Langsten R, Hill K (1998). The accuracy of mothers' reports of child vaccination: evidence from rural Egypt. Soc Sci Med.

[25] George K, Victor S, Abel R (1990). Reliability of mother as an informant with regard to immunisation. Indian J Pediatr.

[26] Valadez JJ, Weld LH (1992). Maternal recall error of child vaccination status in a developing nation. Am J Public Health.

[27] NBS (2013). Tanzania Population and Housing Census: Population Distribution by Administrative Areas 2012.

[28] Binyaruka P, Patouillard E, Powell-Jackson T, Greco G, Maestad O, Borghi J (2015). Effect of paying for performance on utilisation, quality, and user costs of health Services in Tanzania: a controlled before and after study. PLoS One.

[29] Borghi J, Mayumana I, Mashasi I, Binyaruka P, Patouillard E, Njau I, Maestad O, Abdulla S, Mamdani M (2013). Protocol for the evaluation of a pay for performance programme in Pwani region in Tanzania: a controlled before and after study. Implement Sci.

[30] World Bank Impact Evaluation Toolkit [http://web.worldbank.org/WBSITE/EXTERNAL/TOPICS/EXTHEALTHNUTRITIONANDPOPULATION/EXTHSD/EXTIMPEVALTK/0,,contentMDK:23262154~pagePK:64168427~piPK:64168435~theSitePK:8811876,00.html Accessed 9th May 2018].

[31] Viera AJ, Garrett JM (2005). Understanding interobserver agreement: the kappa statistic. Fam Med.

[32] Sim J, Wright CC (2005). The kappa statistic in reliability studies: use, interpretation**,** and sample size requirements. Physical therapy.

[33] Cicchetti DV, Feinstein AR (1990). High agreement but low kappa: II. Resolving the paradoxes. J Clin Epidemiol.

[34] Feinstein AR, Cicchetti DV (1990). High agreement but low kappa: I. The problems of two paradoxes. J Clin Epidemiol.

[35] Byrt T, Bishop J, Carlin JB (1993). Bias, prevalence and kappa. J Clin Epidemiol.

[36] Filmer D, Pritchett LH (2001). Estimating wealth effects without expenditure data--or tears: an application to educational enrollments in states of India. Demography.

[37] Vyas S, Kumaranayake L (2006). Constructing socio-economic status indices: how to use principal components analysis. Health Policy Plan.

[38] Coughlin SS (1990). Recall bias in epidemiologic studies. J Clin Epidemiol.

[39] Altman DG, Bland JM (1994). Diagnostic tests. 1: sensitivity and specificity. Bmj.

